# Guillain-Barré Syndrome Outbreak in Pune, India, Calls for Heightened Awareness and Preparedness

**DOI:** 10.7759/cureus.78609

**Published:** 2025-02-06

**Authors:** Venkataramana Kandi

**Affiliations:** 1 Clinical Microbiology, Prathima Institute of Medical Sciences, Karimnagar, IND

**Keywords:** awareness, guillain-barré syndrome (gbs), intensive care unit (icu), morbidity and mortality, noncommunicable disease, outbreak, treatment and management

## Abstract

The ongoing Guillain-Barré syndrome (GBS) outbreak in Pune, India, has raised significant concerns among public health administrators at both the state and central government levels. Over a hundred cases of GBS have been reported in the region, with some patients being treated in the intensive care unit (ICU) and receiving mechanical ventilation. This outbreak has already resulted in one fatality, and new cases continue to emerge. There is growing concern over a potential rise in cases and the possible spread to other areas. Although GBS is a noncommunicable disease, it is crucial to raise awareness about its causes, clinical features, and treatment and management to mitigate morbidity and mortality associated with this condition.

## Editorial

Guillain-Barré syndrome (GBS) is a rare, debilitating condition that affects the nervous system, typically following bacterial or viral infections. Evidence linking vaccination to GBS confirms the role of microbial components in triggering immune responses that may contribute to the development of GBS. GBS is generally an acute clinical condition, with symptoms lasting hours, days, or weeks and often resolving without significant long-term effects. However, in some cases, GBS can lead to severe complications such as paralysis and death. Patients suffering from GBS experience impairment of the peripheral nervous system, which affects skin sensation and causes muscle weakness, particularly in the legs. This is thought to result from antibodies and other immune cells attacking the nerves, disrupting nerve transmission from the brain and spinal cord.

Electrical signals are transmitted between the brain and the body through the central axon, a component of the nerve responsible for conducting these signals. Furthermore, myelin, the protective covering of a neuron, enables the smooth transmission of electrical signals to distant parts of the body. In GBS, the most common effect on the myelin sheath is acute inflammatory demyelinating polyradiculoneuropathy (AIDP), in which the immune system attacks and destroys the myelin. When axons are damaged in GBS, it leads to acute motor axonal neuropathy (AMAN) and acute motor-sensory axonal neuropathy (AMSAN). In both conditions, immune reactions target the axon, impairing signal transmission and causing muscle weakness and loss of movement. Respiratory muscle failure can occur in severe cases, making breathing difficult, and circulatory collapse may ultimately lead to death.

When GBS affects the cranial nerves that supply the head and neck, it can result in serious complications, including imbalance, loss of coordination, and paralysis. This form of GBS is known as Miller-Fisher syndrome, wherein the patients may experience abnormal reflexes and weakness of the eye and facial muscles. GBS can also involve the autonomic nervous system, affecting patients’ physiological processes like heart rate, blood pressure, respiration, and digestion.

There is no specific age or sex predilection for developing GBS, but generally, individuals over 50 years old may be more predisposed. Interestingly, the exact trigger for GBS remains unclear. While GBS exhibits characteristics of an autoimmune disorder, the specific immune cells responsible for nerve destruction have not yet been identified. It has been observed that molecular mimicry, wherein the microbial components/proteins mimic the nerve tissues, may lead to immune responses that mistakenly attack and destroy the nerves during infections.

Recent respiratory and gastrointestinal infections have been identified as potential triggers for the development of GBS. Gastroenteritis caused by *Campylobacter jejuni* is a well-established trigger. Additionally, infections caused by other viruses, including severe acute respiratory syndrome coronavirus-2 (SARS-CoV-2), norovirus/Norwalk virus, Zika virus, cytomegalovirus (CMV), and Epstein-Barr virus, have also been linked to the onset of GBS.

Lack of awareness among both the public and healthcare professionals can complicate the diagnosis of GBS. Clinical diagnosis of GBS is difficult in the initial stages due to the mild symptoms that are often overlooked, which can lead to delayed diagnosis and hinder effective patient management. It is important for clinicians to carefully assess the history of the patients and look for acute-onset changes in muscle movements, reflexes, and bilateral involvement, key factors that differentiate GBS from other neurological diseases, which are generally chronic. Diagnostic methods that can aid in confirming GBS include nerve conduction studies (nerve conduction velocity (NCV) test), cerebrospinal fluid (CSF) analysis, and magnetic resonance imaging (MRI) of the brain and spinal cord.

Currently, there is no specific treatment modality for managing GBS patients. Management typically involves intensive care unit (ICU) admission, mechanical ventilation, plasmapheresis (separation and replacement of red blood cells (RBCs) and plasma), intravenous immunoglobulin (IVIg), and corticosteroid therapies, which can benefit GBS patients. The potential utility of erythropoietin (EPO), a glycoprotein hormone secreted by the renal tubules that stimulates red blood cell production, has been observed in animal studies, where it showed promise in treating GBS by alleviating autoimmune neuritis and nerve damage. The IgG-degrading enzyme of *Streptococcus pyogenes *(IdeS) has been shown to delay disease progression and reduce symptoms in rabbit models of GBS. Despite the considerable success of treatment with monoclonal antibodies, patients may still be predisposed to developing disabilities or succumb to GBS. Furthermore, the availability of such therapies, particularly in low- and middle-income countries (LMICs), can be limited due to cost and accessibility, further complicating patient management [1,2].

An assessment of GBS in Bangladesh for a period of three years (2010-2013) revealed a high mortality rate of 12% (50/407) among the affected population. Additionally, 48% (24/50) of the patients required mechanical ventilation. This study identified age over 40 years as a risk factor and reported a median time of 18 days from the onset of symptoms to death [3].

A recent study from Ethiopia evaluated the subtypes of GBS based on electrophysiological assessment. The study provided detailed data on clinical manifestations, diagnostic findings, treatment, and prognosis. It revealed that the axonal variant (75.5%) of GBS was the most common, followed by the demyelinating type (24.5%). The mean age of GBS patients was 28.5 years, with the majority (76.7%) falling within the 14-34 age group, and most patients were men (61.7%). Ascending paralysis (76.7%) and reflex abnormalities (91.7%) were the most common clinical presentations. GBS was mostly associated with recent history of gastrointestinal disease (26.7%), upper respiratory tract infections (15%), and vaccination (11.7%). Most patients were treated with IVIgs ( 41.7%), and 26.7% required mechanical ventilation [4].

An outbreak of GBS is currently being experienced in Pune, the second-largest city in Maharashtra, India. As of the most recent update, approximately 110 people have been affected, with the majority being men (68.18%; 75/110). Among the affected population, one patient has died (9.09%; 1/110), and 13 (11.81%) are being treated by mechanical ventilation. Of the 44 stool samples evaluated for enteric viruses, 14 (31.81%) were positive for norovirus, and five (11.36%) were positive for *C. jejuni*. No evidence of dengue, chikungunya, or Zika virus was found among the 59 blood samples tested at the National Institute of Virology (NIV), Pune [5].

The available literature and the ongoing outbreak in India suggest the importance of heightened awareness about GBS. This condition is associated with severe and debilitating neurological complications that can lead to paralysis and death. The significant mortality rate associated with GBS highlights its potential as a serious public health concern. The role of gastrointestinal and respiratory infections as predisposing factors emphasizes the relevance of GBS in regions with poor sanitation and compromised hygiene practices, such as India.
